# Improved YOLOv7-Based Algorithm for Detecting Foreign Objects on the Roof of a Subway Vehicle

**DOI:** 10.3390/s23239440

**Published:** 2023-11-27

**Authors:** Weijun Wang, Jinyuan Chen, Zucheng Huang, Hai Yuan, Peng Li, Xuyao Jiang, Xintong Wang, Cheng Zhong, Qunxu Lin

**Affiliations:** 1 Guangzhou Institute of Advanced Technology, Guangzhou 511458, China; wj.wang@giat.ac.cn (W.W.); jy.chen@giat.ac.cn (J.C.); hai.yuan@giat.ac.cn (H.Y.); xy.jiang@giat.ac.cn (X.J.); xt.wang@giat.ac.cn (X.W.); 2 School of Rail Transportation, Wuyi University, Jiangmen 529020, China; wyi0714lp@163.com (P.L.); zc13257913512@163.com (C.Z.); 13427477615@163.com (Q.L.)

**Keywords:** subway vehicles, foreign roof objects, YOLOv7, Ghost module, WIoU loss function

## Abstract

Subway vehicle roofs must be inspected when entering and exiting the depot to ensure safe subway vehicle operations. This paper presents an improved method for detecting foreign objects on subway vehicle roofs based on the YOLOv7 algorithm. First, we capture images of foreign objects using a line-scan camera at the depot entrance and exit, creating a dataset of foreign roof objects. Subsequently, we address the shortcomings of the YOLOv7 algorithm by introducing the Ghost module, an improved weighted bidirectional feature pyramid network (WBiFPN), and the Wise intersection over union (WIoU) bounding-box regression loss function. These enhancements are incorporated to build the subway vehicle roof foreign object detection model based on the improved YOLOv7, which we refer to as YOLOv7-GBW. The experimental results demonstrate the practicality and usability of the proposed method. The analysis of the experimental results indicates that the YOLOv7-GBW algorithm achieves a detection accuracy of 90.29% at a speed of 54.3 frames per second (fps) with a parameter count of 15.51 million. The improved YOLOv7 model outperforms mainstream detection algorithms in terms of detection accuracy, speed, and parameter count. This finding confirms that the proposed method meets the requirements for detecting foreign objects on subway vehicle roofs.

## 1. Introduction

Subways are the preferred mode of transportation for the public [1]. However, when foreign objects land on the roofs of subway vehicles, it can lead to serious subway operation accidents. The condition of every subway vehicle roof must be inspected as it enters and exits the depot to prevent accidents caused by foreign object intrusion on subway vehicle roofs.

The traditional status detection for subway vehicle roofs primarily relies on manual inspection [2]. However, manual inspection is prone to inefficiency, high labor intensity, and the risk of overlooking secondary foreign objects [3]. To address the shortcomings of manual inspection, researchers have begun to explore traditional image processing detection methods based on template matching [4]. For instance, Lin [5] employed invariant moment features for the detection and recognition of foreign objects on locomotive roofs using feature-based matching methods, and Cao [6] used an improved image difference algorithm to detect foreign objects on locomotive roofs. While these template-matching-based traditional image processing detection methods have displayed some detection capabilities, they generally suffer from low accuracy, slow processing speeds, and high computational parameter counts. These methods struggle to meet the rapid and high-precision detection requirements for subway vehicle roofs. However, detection methods based on deep learning [7] have the potential to address these limitations.

Deep learning offers end-to-end detection capabilities with strong feature learning capabilities and high efficiency. Qian et al. [8] proposed that methods using deep learning for locomotive roof detection would become mainstream with the continuous development of deep learning. Wang [9] used an improved YOLOv4 algorithm [10] for feature extraction and feature fusion networks to detect foreign objects in the pantograph area of light rail trains. The improved YOLOv4 algorithm achieved a detection accuracy of 82.6% and a detection speed of 22.3 fps, demonstrating the feasibility of deep learning-based detection methods.

Therefore, referring to Wang Chensong’s method, aiming at the problems of many types of foreign bodies on the roof of metro vehicles and different sizes of foreign bodies, this paper uses the currently collected images of foreign bodies on the roof of metro vehicles as the training dataset of the algorithm model and uses the YOLOv7 algorithm with better overall performance. Then, according to the requirements of rapid and high-precision detection of the railway vehicle roof, the YOLOv7 algorithm is improved, and the improved yolov7 algorithm model is named the YOLOv7-GBW algorithm model.

The main improvements in this paper are as follows:

1. Integration of the Ghost Module: Drawing inspiration from the GhostNet architecture, the standard convolutions in the YOLOv7 algorithm model are replaced with Ghost modules. This modification makes the YOLOv7 algorithm model lighter and enhances its detection accuracy.
2. Enhanced Feature Fusion: Addressing the problem of insufficient feature fusion information in the YOLOv7 algorithm model, this paper borrows from the weighted bidirectional feature pyramid network (WBiFPN) to optimize feature fusion. This optimization is achieved without significantly increasing the parameter count while making the feature fusion capabilities of the YOLOv7 algorithm model more comprehensive.
3. Wise Intersection over Union Loss (WIoU) Function: This paper replaces the original complete intersection over union (CIoU) bounding-box loss function of the YOLOv7 algorithm model with the WIoU bounding-box loss function. This approach further improves the bounding-box regression accuracy and detection precision of the YOLOv7 algorithm model.

These improvements collectively aim to enhance the performance of the YOLOv7 algorithm for foreign object detection on subway vehicle roofs, making it more lightweight, accurate, and capable of handling diverse object categories and sizes.

## 2. Subway Vehicle Roof Foreign Object Detection Algorithm Based on YOLOv7-GBW

### 2.1. YOLOv7 Algorithm

**Figure 1:**
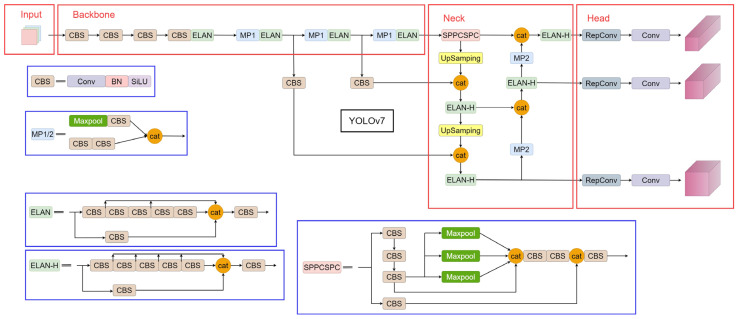
YOLOv7 algorithm model structure.

The YOLOv7 algorithm model [11] is the latest addition to the YOLO series of object detection algorithms developed by the YOLOv4 team [10], offering faster detection speeds and higher accuracy than most existing object detection algorithm models with detection rates ranging from 5 to 160 fps. Figure 1 illustrates the structure of the YOLOv7 algorithm model.

As depicted in Figure 1, the YOLOv7 algorithm model comprises four main components: image input, backbone, neck, and head. The backbone incorporates the CBS module, efficient layer aggregation network (ELAN) module, and MP1 convolution module. The CBS module is a regular convolutional module, while ELAN is an efficient layer aggregation network proposed for the YOLOv7 algorithm model. Through expansion and transformation, it enhances the learning performance of the algorithm model without affecting the original gradient path, thereby increasing the computational capability of the algorithm model in terms of parameters. The MP1 convolutional module is added after the CBS module, forming upper and lower branches with the addition of a maxpool layer. Concatenation is then applied to integrate the features from the two branches, enhancing the feature extraction performance of the feature extraction network.

To facilitate different input sizes, YOLOv7 replaces the SPP (spatial pyramid pooling) module in the neck with the SPPCSPC module, which is also a variant of the spatial pyramid pooling module. Its advantages include reducing image distortion caused by image processing and addressing the problem of feature redundancy extraction during convolution. Furthermore, it adopts the same aggregated feature pyramid network (PAFPN) structure as the YOLOX algorithm model published by Megvii Research in 2021. This structure allows for deep-level features to be transmitted from bottom to top for inter-layer feature fusion. The neck network also incorporates the ELAN-H module and the MP2 module. The ELAN-H module is wider than the ELAN module, and the primary difference between the MP2 and MP1 modules lies in the number of channels with identical structures. Finally, the head network employs the RepConv module, combining reparameterized convolutions with the model network structure to strike a balance between speed and accuracy during the training process.

### 2.2. Ghost Module

To reduce the parameter count of the YOLOv7 algorithm model and enhance its detection speed, we introduce the Ghost module from GhostNet [12] to replace the CBS module, resulting in the Ghost-based (GBS) module. GhostNet is a lightweight algorithm model developed by Huawei, and its Ghost module plays a pivotal role in achieving these improvements.

**Figure 2:**
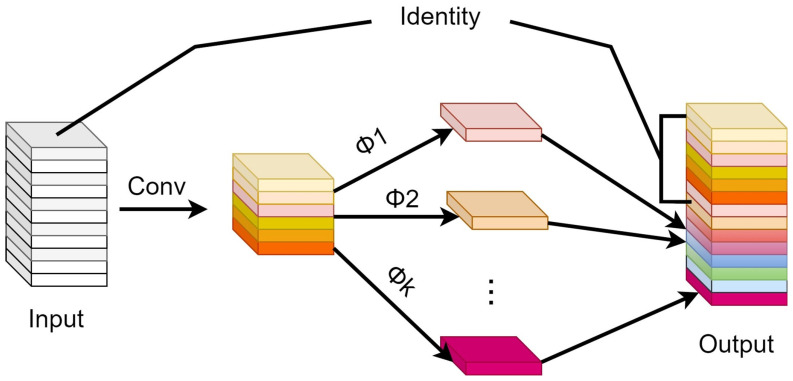
Ghost module structure diagram.

The Ghost module, as implemented in GhostNet, achieves a significant parameter reduction while preserving essential information features through a series of simple linear transformations. This approach enables the generation of critical feature maps with minimal computational overhead. Figure 2 illustrates the structure of the Ghost module.

In regular convolutions, the output feature maps often contain considerable redundant information with many features being similar. To reduce the redundancy introduced by conventional convolutions, the Ghost module in GhostNet employs straightforward linear transformations on a small subset of the original feature maps to obtain numerous feature maps that are functionally equivalent to those produced by standard convolutions. This process is represented by Equation (1).

(1) $$y_{i,j}=\Phi_{i,j}(y_{i}^{\prime },\forall i=1,\ldots ,m;j=1,\ldots ,s),$$

where $y^{\prime }$ represents a small subset of the original feature maps of Y (feature maps obtained through conventional convolution), ranging from the *i*th to the (*i+k*)th feature map. In addition, $\Phi_{i,j}$ is a method function used to generate the linearly transformed feature maps. This approach allows for the Ghost module to generate feature maps with reduced redundancy and computational cost while maintaining the essential information needed for effective feature learning.

The number of parameters involved in a regular convolution is given by Equation (2), assuming an input feature map size of h×w, output feature map size of $h^{\prime }\times w^{\prime }$, and convolutional kernel with n filters, c channels, and a kernel size of k×k. For the Ghost module with linear transformation convolutional kernels of size d×d and an acceleration factor of s, the number of parameters involved is calculated using Equation (3). The computation compression ratio $r_{c}$, as indicated in Equation (4), is approximately equal to the specified acceleration factor, indicating that the Ghost module significantly reduces the computational complexity compared to regular convolutions through simple linear operations while only marginally affecting the detection speed of the algorithm model. This approach leads to a significant improvement in the detection accuracy of foreign objects on subway vehicle roofs.

(2) $$S1=h^{\prime }\times w^{\prime }\times n\times k\times k\times c,$$

(3) $$S2=h^{\prime }\times w^{\prime }\times \frac{n}{s}\times k\times k\times c+(s-1)\times h^{\prime }\times w^{\prime }\times \frac{n}{s}\times k\times k,$$

(4) $$r_{c}=\frac{S1}{S2}=\frac{n\times c}{\frac{n}{x}\times c+(s-1)\times \frac{n}{s}}=\frac{s\times c}{c+s-1}\approx s,$$

### 2.3. BiFPN Algorithm

**Figure 3:**
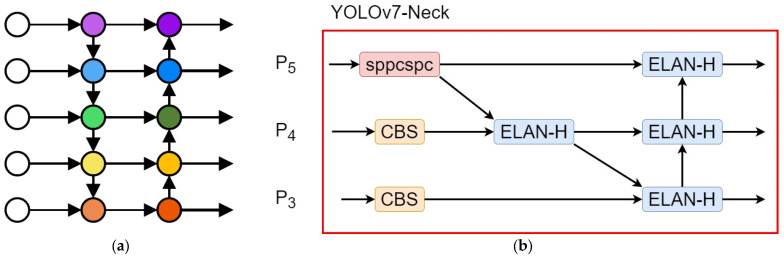
(**a**) Path aggregation network structure and (**b**) YOLOv7-neck. CBS: Conv BN SiLU, ELAN-H: efficient layer aggregation network-H, SPPCSP: spatial pyramid pooling cross-stage partial channel.

The neck network used in the YOLOv7 algorithm model employs the path aggregation network (PANet) structure, integrating features from different hierarchical levels through a bidirectional network operating from top to bottom and bottom to top. The neck network in the YOLOv7 algorithm model enhances the PANet structure by omitting the upsampling layer, downsampling layer (MP2 module), and concatenation layer (Concat). Consequently, the neck network in the YOLOv7 algorithm model represents an improved version of the PANet, as illustrated in Figure 3.

Figure 3b reveals that the neck network of the YOLOv7 algorithm model, based on the PANet, improves cross-level feature fusion. The SPP module with a cross-stage partial channel module in the P_5_ layer in Figure 3 passes features down to the middle ELAN-H module in the P_4_ layer, which transfers features to the rightmost ELAN-H module in the P_3_ layer, ensuring more comprehensive feature fusion.

To further enhance feature fusion performance in the YOLOv7 algorithm model, this paper draws inspiration from the BiFPN for weighted bidirectional feature pyramid fusion, offering a faster and more lightweight approach [13]. The BiFPN represents another type of feature fusion network, building upon the feature pyramid network structure, similar to PANet.

**Figure 4:**
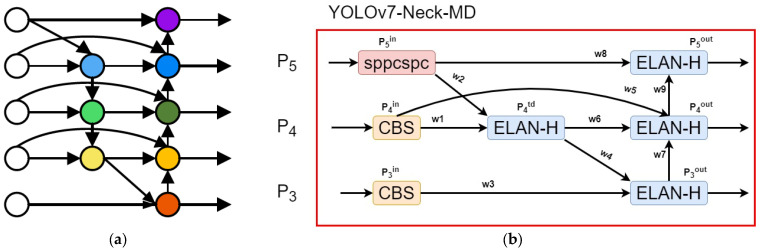
(**a**) Bidirectional feature pyramid network (BiFPN) and (**b**) WBiFPN.

Moreover, BiFPN reconfigures the top-down and bottom-up channels outside the forward propagation and introduces horizontal connections between features of the same size, addressing the information loss caused by excessive layer depth. Accordingly, the neck network in the YOLOv7 algorithm model improved, resulting in an enhanced WBiFPN neck network, as depicted in Figure 4.

Figure 4b clearly illustrates that the improvement strategy draws inspiration from the BiFPN. In the middle layer (*P*_4_), a horizontal connection was added to link the output of the CBS module to the ELAN-H module. This enhancement enables the neck network to fuse more features, further enhancing its ability to capture fine-grained details and improving the detection accuracy of the YOLOv7 algorithm model.

In the WBiFPN structure, *P_3_* to *P_5_* represent different feature inputs passed from the backbone network. Each output is defined in Equations (5)–(8).

(5) $$P_{3}^{out}=Conv(\frac{\omega_{3}*P_{3}^{in}+\omega_{4}*uSample(P_{4}^{td})}{\omega_{3}+\omega_{4}+\varepsilon }),$$

(6) $$P_{4}^{td}=Conv(\frac{\omega_{1}*P_{4}^{in}+\omega_{2}*uSample(P_{5}^{in})}{\omega_{1}+\omega_{2}+\varepsilon }),$$

(7) $$P_{4}^{out}=Conv(\frac{\omega_{5}*P_{4}^{in}+\omega_{6}*P_{4}^{td}+\omega_{1}*dSample(P_{3}^{out})}{\omega_{5}+\omega_{6}+\omega_{7}+\varepsilon }),$$

(8) $$P_{5}^{out}=Conv(\frac{\omega_{8}*P_{5}^{in}+\omega_{9}*dSample(P_{4}^{out})}{\omega_{8}+\omega_{9}+\varepsilon }),$$

Within these equations, $P_{i}^{in}$ represents the incoming features, $P_{i}^{out}$ represents the features after fusion and outflow, uSample denotes upsampling features, dSample denotes downsampling features, $\omega_{i}$ represents the weights of various feature flow paths, and ε is a small constant 1 × 10^−5^ used to prevent the generation of unstable extreme values during the feature flow process.

These equations describe the flow of features within the WBiFPN structure and how various operations are applied to achieve feature fusion and flow within the network. The weights and constants are critical in controlling and stabilizing the feature flow process.

### 2.4. WIoU Boundary-Box Loss Function

The original YOLOv7 algorithm model employed the CIoU boundary-box regression loss function. The CIoU loss function includes a penalty for the aspect ratio, which becomes ineffective when the aspect ratio of the predicted bounding box matches that of the ground truth bounding box, leading to instability in the CIoU loss function.

This paper introduces an improved WIoU boundary-box loss function with a dynamic nonmonotonic focus mechanism to address this problem. This enhancement ensures the stability and effectiveness of the loss function, even when the aspect ratios of the predicted and ground truth bounding boxes are equal. The dynamic nonmonotonic focus mechanism likely enables the model to adapt better to various scenarios and object shapes, enhancing the accuracy and stability of the object detection capabilities of the YOLOv7 algorithm model.

The WIoU loss function, specifically WIoU-v3 [14], incorporates an innovative, dynamic, nonmonotonic focus mechanism that employs outliers to assess the quality of anchor boxes and allocate gradient gains more effectively. This mechanism reduces the competitiveness of high-quality anchor boxes while mitigating the harmful gradients caused by low-quality ones. It allows for the WIoU loss function to focus on both standard anchor boxes and improve the performance of the algorithm model.

The WIoU loss function has undergone three iterative versions, and this paper references WIoU-v3; its expressions are illustrated in Equations (9) to (11). In Equation (11), r represents a non-monotonic focusing coefficient. In Equation (10), $L_{WIoUv1}$ is constructed based on distance metrics to build distance attention and achieve the two-layer attention mechanism in the WIoU-v1 version. $L_{IoU}$ is used to measure the overlap between predicted boxes and ground truth boxes in object detection tasks. In Equation (9), $W_{g}$ and $H_{g}$, respectively, denote the width and height of the minimum bounding box, while x and y represent the coordinates of the predicted box, and $x_{gt}$ and $y_{gt}$ denote the coordinates of the ground truth box. To prevent the impact of gradients on the convergence of $R_{WIoU}$, $W_{g}$ and $H_{g}$ are detached from the computation graph in Equation (9), indicated by the superscript ‘*’. This action effectively eliminates factors that could affect convergence, and hence, no new variables are introduced. While $R_{WIoU}\in \left.[1,e\right)$ will noticeably expand $L_{IoU}$ for anchor boxes of general quality, $L_{IoU}\in \left[0,1\right]$ will also significantly reduce $R_{WIoU}$ for high-quality anchor boxes. Moreover, when anchor boxes overlap with target boxes, $L_{IoU}$ will prioritize the variation in the distance between their center points.

(9) $$R_{WIoU}=\exp(\frac{(x-x_{gt}{)}^{2}+(y-y_{gt}{)}^{2}}{(W_{g}^{2}+H_{g}^{2}{)}^{*}})$$

(10) $$L_{WIoUv1}=R_{WIoU}L_{IoU}$$

(11) $$L_{WIoUv3}=rL_{WIoUv1}$$

The additional computational cost introduced by the WIoU loss function primarily arises from the calculation of focus coefficients and the averaging of IoU losses. Under the same experimental conditions, WIoU is faster than CIoU because it does not require the calculation of aspect ratios. The computational time for WIoU is 87.2% of that for CIoU.

Tong et al. demonstrated the effectiveness of applying the WIoU loss function to the state-of-the-art YOLOv7 algorithm model. When used with the MS-COCO dataset, it improved the average precision at an IoU threshold of 0.75 from 53.03% to 54.50%. This outcome illustrates the positive effect of the WIoU boundary-box loss function on enhancing the performance of the YOLOv7 algorithm model.

## 3. Experiment

### 3.1. Dataset Preparation

**Figure 5:**
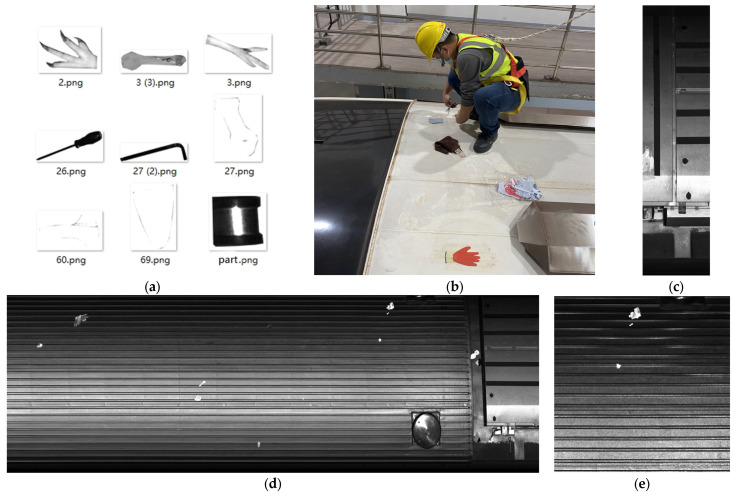
Process of creating images of foreign objects on subway vehicle roofs: (**a**) Samples of collected foreign objects; (**b**) Manual affixing of foreign objects; (**c**) Small images captured with line-scan camera; (**d**) Stitched complete image; (**e**) Cropped roof foreign object image.

Since publicly available datasets containing images of foreign objects on subway vehicle roofs were unavailable, a significant number of such images were quickly acquired using the following procedure. First, photographs of foreign objects manually detected on Line 2 of Xi’an Metro, Line 2 of Dongguan Metro, and Line 7 of Shenzhen Metro were taken. These photographs were printed in color on paper. Subsequently, all collected foreign objects were cropped based on their contours, as demonstrated in Figure 5a. Next, the cropped paper pieces featuring various foreign objects were randomly affixed to the roof of a subway vehicle on Line 2 of Xi’an Metro (Figure 5b). Images of the vehicle roof were captured as the subway vehicles passed through the gantry crane at the entry and exit of the subway vehicle depot using a line-scan camera mounted on top of the gantry crane. Using speed radar devices positioned on overhead gantries, real-time measurements of subway train speeds are taken to ensure that the camera’s capture frequency is synchronized with the train’s speed. This process resulted in numerous small roof images (Figure 5c). Finally, the collected small images were stitched together in equal quantities to ensure that most areas of the vehicle roof were visible (Figure 5d). The large, stitched image was cropped into 640 × 640 segments (Figure 5e). This process yielded a total of 3568 images featuring foreign roof objects.

After obtaining these roof images with foreign objects, they were annotated using annotation software (MVTec Deep Learning Tool, v 22.10.0.0). Data augmentation was applied in three ways, spatial transformations, adding noise, and color transformations, to prevent overfitting during the model training process, as described in several studies [15,16,17]. After data augmentation, the total number of roof images with foreign objects increased to 11,689. Data augmentation improved the model generalization and enhanced its training effectiveness.

The dataset was formatted according to the standard dataset format for foreign objects on subway vehicle roofs. Finally, the dataset was divided into training, validation, and testing sets in a 7:2:1 ratio. With this, the creation of the foreign object dataset for subway vehicle roofs was completed.

### 3.2. Experimental Environment

The hardware configuration for the experimental platform in this paper primarily includes an Intel i9-12900K CPU processor, 64 GB of RAM, and an NVIDIA RTX 3090 GPU graphics card with 24 GB of VRAM. Additionally, an environment for training and simulation experiments was established on a Dell Precision 3660 workstation comprising the following components:

- Ubuntu 20.04 operating system;
- CUDA 11.6;
- Python 3.9;
- PyTorch 1.12.1;
- Various dependent libraries.

This environment was used to conduct experiments and simulations.

### 3.3. Evaluation Metrics and Training Parameters

Before training the algorithm model, the evaluation metrics must be defined, and the training parameters must be initialized. In terms of the evaluation metrics, this paper selected six metrics: the mean average precision at IoU 0.5 (mAP0.5), precision, recall, fps, parameter count, and floating-point operations per second (FLOPS). Table 1 provides the specific settings for initializing the training parameters. Setting hyperparameters is a crucial task that can impact the performance of a model and the effectiveness of algorithm improvements. When making improvements to the YOLOv7 algorithm model, maintaining consistency in hyperparameter settings is of utmost importance. On one hand, keeping hyperparameter settings consistent ensures effective algorithm enhancements. By maintaining consistent hyperparameters, it becomes easier to compare algorithm performance before and after improvements and accurately assess the effects of these enhancements. If hyperparameters are changed during the improvement process, it becomes challenging to distinguish whether the performance changes are due to the algorithm’s inherent improvements or the variations in hyperparameters. Therefore, in this paper, we have chosen to keep the hyperparameter settings consistent with the YOLOv7 algorithm model.

**Figure 6:**
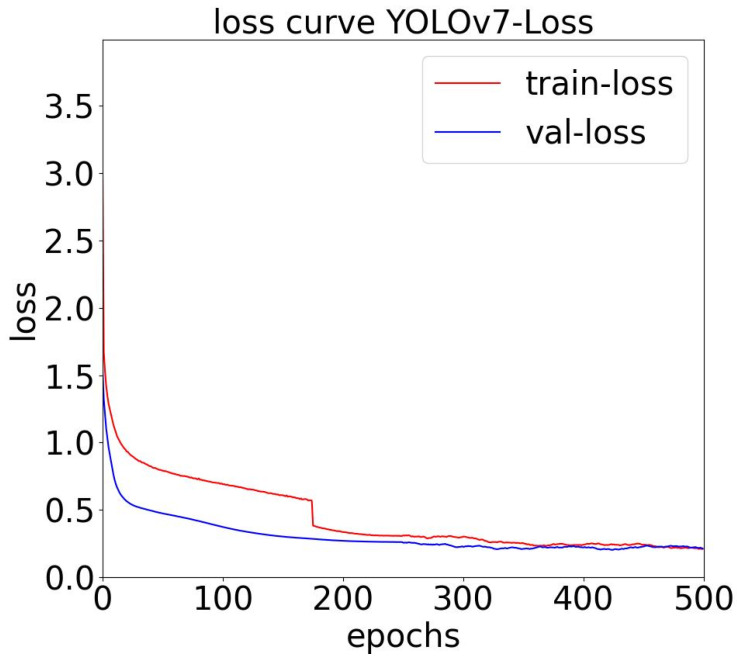
Training loss curve and verifying loss curve.

Figure 6 indicates that the final loss values for the training and validation sets were 0.03 and 0.026, respectively, for the YOLOv7 algorithm model to ensure that the roof foreign object dataset did not experience overfitting during training.

## 4. Experimental Result Analysis

### 4.1. Analysis of Adding the Ghost Module

To better assess the effectiveness of the GBS module in enhancing the detection accuracy of the YOLOv7 algorithm model, this paper conducted experiments by replacing CBS modules in two areas of the YOLOv7 algorithm model: the backbone and neck. The modified models were designated as YOLOv7-G-B (backbone), YOLOv7-G-N (neck), and YOLOv7-G-BN (backbone and neck). To ensure the validity of the experiments, all data results have undergone no fewer than 10 repetitions of training and validation experiments. The best and worst results are excluded, and the remaining experimental values are averaged to obtain the experimental outcome. Table 2 details the results following the experimental training and validation.

Table 2 reveals that the YOLOv7-G-BN algorithm model outperforms the other three models. It demonstrates the most substantial improvements in mAP0.5, precision, and recall with each metric improving by approximately 2.5%. Furthermore, it has the lowest parameter count and FLOPS among the models. While the YOLOv7-G-BN algorithm model’s detection speed in frames per second (FPS) experiences a certain decrease due to the additional computational overhead introduced by the Ghost module, resulting in an FPS of only 48.7, the YOLOv7-G-BN algorithm model can still meet real-time detection requirements. Moreover, considering its detection accuracy and parameter quantity, this improvement approach demonstrates a certain level of effectiveness. Adding the Ghost module to the backbone and neck networks of the YOLOv7 algorithm model has indeed shown the potential to improve detection accuracy while reducing the model’s parameter count. This can be a significant achievement in terms of optimizing object detection models, making them more efficient and effective for various applications.

### 4.2. Experimental Analysis of the Improved Feature Fusion Network

This paper presents experiments using the dataset with the original YOLOv7 algorithm model and YOLOv7-B model to evaluate the performance of adding the three-layer WBiFPN network to the neck network of the original YOLOv7 algorithm model (YOLOv7-B). To ensure the validity of the experiments, all data results have undergone no fewer than 10 repetitions of training and validation experiments. The best and worst results are excluded, and the remaining experimental values are averaged to obtain the experimental outcome. Table 3 presents the detailed results.

Table 3 reveals that, compared to the original YOLOv7 algorithm model, the YOLOv7-B algorithm model demonstrates an increase of approximately 1.5% in mAP0.5, a slight decrease of 2% in precision, and an increase of approximately 4% in recall. The parameter count, FLOPS, and detection speed (fps) all experience slight increases. This outcome demonstrates that the WBiFPN provides improved feature fusion for the input features of the feature extraction network, further enhancing the mAP0.5 and recall of the algorithm model.

### 4.3. Experimental Analysis of Adding the WIoU Loss Function

This study compares the training losses of the original YOLOv7 algorithm model with the improved YOLOv7 algorithm model (YOLOv7-W) to assess the effectiveness of replacing the CIoU bounding-box loss function with the WIoU bounding-box loss function on the performance of the YOLOv7 algorithm model. Experiments were conducted using the foreign object dataset, and the detailed results are presented in Table 4. To ensure the validity of the experiments, all data results have undergone no fewer than 10 repetitions of training and validation experiments. The best and worst results are excluded, and the remaining experimental values are averaged to obtain the experimental outcome.

**Figure 7:**
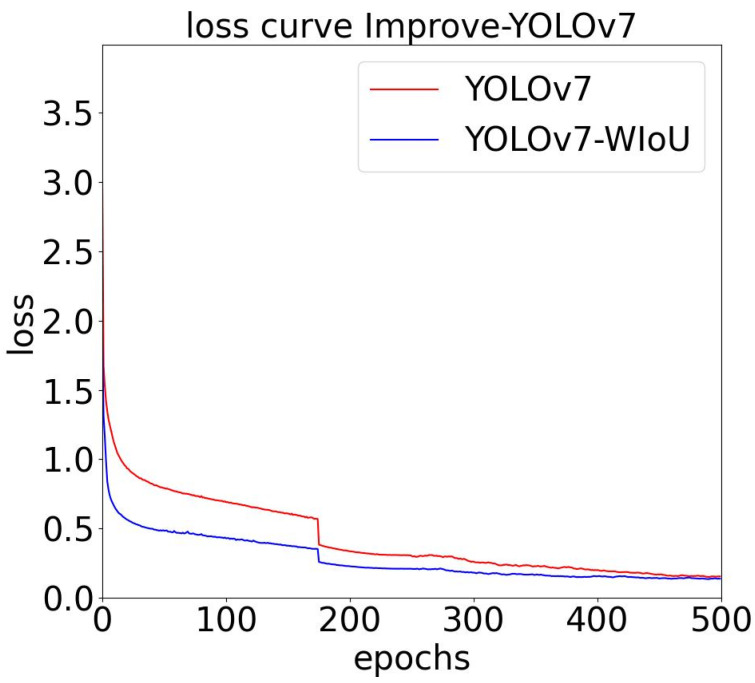
Training loss curve and verifying loss curve.

Figure 7 illustrates that replacing the CIoU bounding-box loss function with the WIoU bounding-box loss function significantly enhances the training convergence speed of the YOLOv7 algorithm model, converging to approximately 0.021. This outcome indicates that the replacement has a noticeable acceleration effect on training.

In Table 4, in comparison to the original YOLOv7 algorithm model, the YOLOv7-W algorithm model demonstrates an increase of approximately 2.5% in mAP0.5, approximately 1.5% in precision, and approximately 2% in recall. The parameter count and FLOPS remain unchanged with a slight improvement in detection speed (fps). In summary, under nearly identical model parameters, computational FLOPS, and detection speed (fps), the YOLOv7-W algorithm model achieves higher detection accuracy and converges faster during training. This result underscores the notable performance improvement provided by the WIoU bounding-box loss function for the YOLOv7 algorithm model.

### 4.4. Overall Analysis

#### 4.4.1. Ablation Experiments

In this study, three improvements were proposed: the Ghost module (YOLOv7-G-BN to YOLOv7-G conversion), the WBiFPN, and the WIoU bounding-box loss function. Different ablation experiments were conducted under the same experimental settings to examine the individual effects and effectiveness of these three improvement methods. These experiments aimed to investigate the differences between the original YOLOv7 algorithm model and various improvement strategies, including the addition of one, two, or all three optimization methods. To ensure the validity of the experiments, all data results have undergone no fewer than 10 repetitions of training and validation experiments. The best and worst results are excluded, and the remaining experimental values are averaged to obtain the experimental outcome. Table 5 presents the results of these ablation experiments.

Table 5 reveals that, compared to the YOLOv7 algorithm model, the YOLOv7-GBW algorithm model exhibits a 4% improvement in mAP0.5, an approximately 2.5% increase in precision, and an approximately 6% increase in recall. Additionally, the model experiences a reduction in parameters of approximately 60%, a decrease in FLOPS by approximately 63%, and only a minor decrease of 10 fps in detection speed. It is evident that the YOLOv7-GBW algorithm model significantly outperforms the YOLOv7 algorithm model in terms of computational complexity and spatial complexity. These results demonstrate that the YOLOv7-GBW algorithm model, which incorporates all three improvement methods, represents the optimal algorithm model.

#### 4.4.2. Comparison with Other State-of-the-Art Object Detection Algorithm Models

To further validate that the proposed YOLOv7-GBW algorithm model is the best-performing model, we conducted comparative experiments in the same experimental environment with five other mainstream algorithm models: the YOLOv7 algorithm model, faster region-based convolutional neural network (R-CNN) algorithm model [18], single-shot detector (SSD) algorithm [19], YOLOv5s algorithm model [20], and YOLOXs algorithm model [21]. To ensure the validity of the experiments, all data results have undergone no fewer than 10 repetitions of training and validation experiments. The best and worst results are excluded, and the remaining experimental values are averaged to obtain the experimental outcome. Table 6 lists the experimental results.

**Figure 8:**
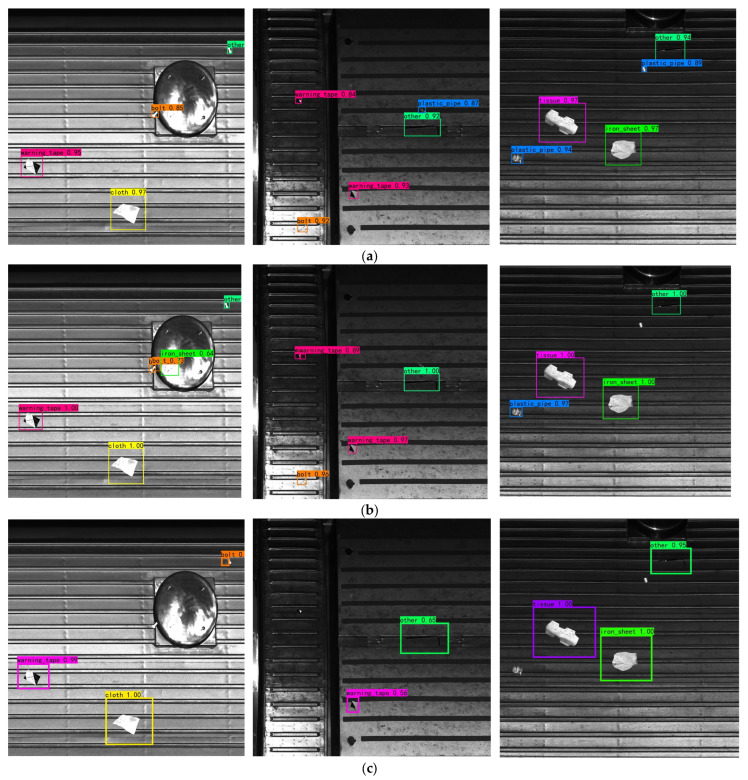

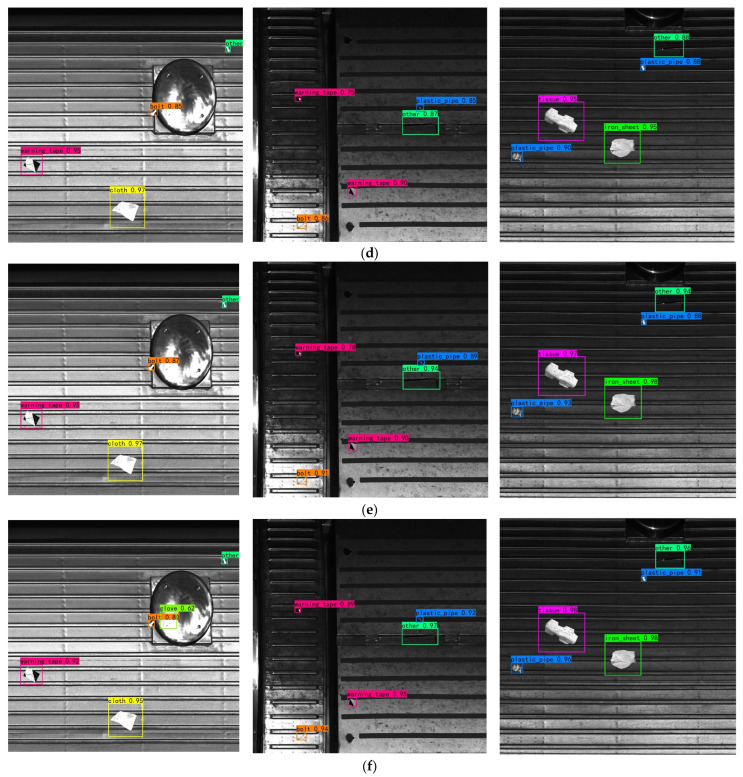
Comparison of six algorithm models: (**a**) YOLOv7, (**b**) Faster-RCNN, (**c**) SSD, (**d**) YOLOv5s, (**e**) YOLOXs, and (**f**) YOLOv7-GBW.

Table 6 demonstrates that the YOLOv7-GBW algorithm model has a clear advantage over the other five mainstream algorithm models in terms of three evaluation metrics: mAP0.5, precision, and recall. Compared to the SSD algorithm, the method proposed in this paper achieves a 26% improvement in mAP0.5 while maintaining a model size of approximately half that of the SSD algorithm model. This result indicates a significant advantage in detection accuracy. Furthermore, compared to the YOLOv5s algorithm model and YOLOXs algorithm model, both of which have half the volume of the YOLOv7-GBW algorithm model, the YOLOv7-GBW algorithm model remains relatively lightweight and demonstrates a substantial improvement in detection accuracy. Additionally, its detection speed (fps) is sufficient for real-time detection requirements, confirming that the YOLOv7-GBW algorithm model proposed in this paper is the optimal algorithm model. Figure 8 depicts the detection results of the six algorithm models.

The comparative graph in Figure 8 highlights that the other five algorithm models all suffer from the problem of missed detections when dealing with small objects as anomalies. In this study, small objects are defined as those with dimensions smaller than 32 × 32 pixels, following the standard criteria used in the field of object detection, as exemplified by the COCO dataset. Medium-sized objects are defined as objects with dimensions ranging from 32 × 32 to 96 × 96 pixels, whereas large objects are those larger than 96 × 96 pixels.

**Figure 9:**
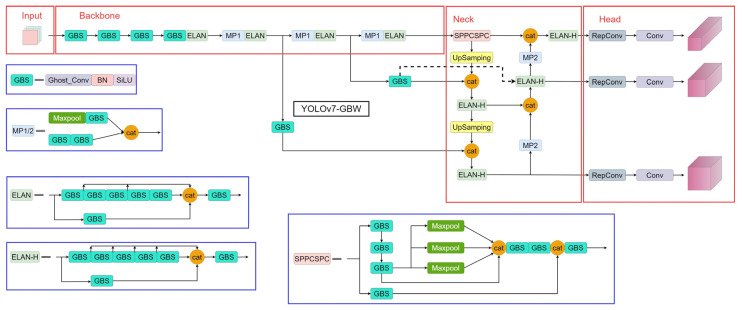
YOLOv7-GBW algorithm model structure.

Furthermore, when the vehicle roof components are significantly dirty, the SSD algorithm model provides false positives. However, the YOLOv7-GBW algorithm model proposed in this paper accurately detects and identifies all roof anomalies and demonstrates better bounding-box regression performance and higher confidence compared to the YOLOv7 algorithm model. This result further underscores the suitability of the YOLOv7-GBW algorithm model as the best choice for detecting anomalies on subway vehicle rooftops. The overall framework of the YOLOv7-GBW algorithm model is shown in Figure 9.

## 5. Conclusions

In this study, we proposed an improved YOLOv7-GBW algorithm model based on the YOLOv7 algorithm model for the task of detecting anomalies on subway vehicle rooftops. First, to avoid making the improved model excessively large, we replaced some convolutional layers with Ghost convolutions. This approach significantly reduced the model size and computational parameters while enhancing recognition accuracy. Second, to improve the ability of the model to extract features at different scales and reduce feature map losses, we replaced the pyramid aggregation feature pyramid structure in the neck network of the original YOLOv7 algorithm model with an improved BiFPN (WBiFPN). This replacement increased the model inference speed and significantly improved its recognition accuracy. Additionally, to further enhance the bounding-box regression capability, we introduced the WIoU bounding-box loss function in the head network of the YOLOv7 algorithm model. This approach led to a further improvement in the overall mAP0.5 value, achieving a recognition accuracy of 90.29%.

We compared the YOLOv7-GBW algorithm model with the five mainstream algorithm models: the YOLOv7, Faster R-CNN, SSD, YOLOv5s, and YOLOXs algorithm models; the YOLOv7-GBW algorithm model outperformed the others in multiple metrics, such as mAP0.5, precision, recall, parameter count, and computational FLOPS. Moreover, it maintained superior performance in detection speed (fps), improving the model.

Applying the YOLOv7 algorithm model to the detection of anomalies on subway vehicle rooftops meets practical requirements. However, this algorithm still has some limitations. The images are single-channel grayscale images, which do not capture as many anomaly features as color images. Additionally, the algorithm does not consider the influence of special cases, such as overlapping and intersections of anomalies, on detection accuracy. Future research will focus on addressing these shortcomings and further optimizing the algorithm to enhance its robustness.

## Figures and Tables

**Table 1:** Initialization of training parameters for the algorithm model.

Parameter Name	Parameter Value
Initial learning rate (Init_Lr)	0.02
Minimum learning rate (Min_Lr)	0.0002
Learning rate decay type (Lr_Decay_Type)	Cosine
Total training epochs (Total_Epochs)	500
Batch size per training iteration (Batch_Size)	16
Network architecture optimization algorithm (Optimizer_Type)	SGD
Momentum for optimization (Momentum)	0.937
Weight decay coefficient (Weight_Decay)	0.0002

**Table 2:** Ghost module experiment verification.

Algorithm	mAP0.5	Precision	Recall	Parameter Count	FLOPS	fps
YOLOv7	86.09%	91.22%	81.72%	37.25 M	108.33 G	64.5
YOLOv7-G-B	87.77%	91.32%	83.49%	26.75 M	54.53 G	54.6
YOLOv7-G-N	88.24%	91.71%	84.83%	24.37 M	86.43 G	53.5
YOLOv7-G-BN	88.59%	92.37%	85.29%	13.86 M	35.66 G	48.7

**Table 3:** Improved YOLOv7 neck network.

Algorithm	mAP0.5	Precision	Recall	Parameter Count	FLOPS	fps
YOLOv7	86.09%	91.22%	81.72%	37.25 M	108.33 G	64.5
YOLOv7-B	87.35%	89.07%	85.52%	39.69 M	117.57 G	68.1

**Table 4:** Improved YOLOv7 algorithm model boundary frame loss function.

Algorithm	mAP0.5	Precision	Recall	Parameter Count	FLOPS	fps
YOLOv7	86.09%	91.22%	81.72%	37.25 M	108.33 G	64.5
YOLOv7-W	88.42%	92.57%	83.88%	37.25 M	108.33 G	64.6

**Table 5:** Comparison of ablation experiments of the three improved methods.

Algorithm	mAP0.5	Precision	Recall	Parameter Count	FLOPS	fps
YOLOv7	86.09%	91.22%	81.72%	37.25 M	108.33 G	64.5
YOLOv7-G	88.59%	92.37%	85.29%	13.86 M	35.66 G	48.7
YOLOv7-B	87.35%	89.07%	85.52%	39.69 M	117.57 G	68.1
YOLOv7-W	88.42%	92.57%	83.88%	37.25 M	108.33 G	64.6
YOLOv7-GB	89.02%	92.67%	86.77%	15.51 M	42.29 G	54.3
YOLOv7-GW	89.47%	92.01%	85.01%	13.86 M	35.66 G	48.7
YOLOv7-BW	89.07%	91.83%	86.87%	38.34 M	110.74 G	65.6
YOLOv7-GBW	90.29%	93.67%	87.56%	15.51 M	42.29 G	54.3

**Table 6:** Performance comparison of six algorithm models.

Algorithm	mAP0.5	Precision	Recall	Parameter Count	FLOPS	fps
YOLOv7	86.09%	91.22%	81.72%	37.25 M	108.33 G	64.5
Faster-RCNN	85.03%	88.45%	78.05%	43.96 M	210.06 G	5.2
SSD	65.11%	76.27%	43.66%	25.22 M	61.95 G	60.6
YOLOv5s	76.86%	84.13%	68.83%	7.10 M	16.57 G	73.6
YOLOXs	79.32%	82.43%	74.33%	8.94 M	26.78 G	180
YOLOv7-GBW	90.29%	93.67%	87.56%	15.51 M	42.29 G	54.3

## Data Availability

Data are contained within the article.
